# Role of Goats in the Epidemiology of *Coxiella burnetii*

**DOI:** 10.3390/biology11121703

**Published:** 2022-11-25

**Authors:** Sofia Anastácio, Sérgio Ramalho de Sousa, Maria José Saavedra, Gabriela Jorge da Silva

**Affiliations:** 1 Vasco da Gama Research Centre (CIVG), Department of Veterinary Sciences, Vasco da Gama University School, Avenida José R. Sousa Fernandes 197 Lordemão, 3020-210 Coimbra, Portugal; 2 Center of Neurosciences and Cell Biology, Health Science Campus, 3000-548 Coimbra, Portugal; 3 Laboratory Medical Microbiology—Antimicrobials, Biocides and Biofilms Unit, Department of Veterinary Sciences, School of Agrarian and Veterinary Sciences, University of Trás-os-Montes e Alto Douro, Quinta de Prados, 5000-801 Vila Real, Portugal; 4 Centre for the Research and Technology Agro-Environmental and Biological Sciences and Inov4Agro—Institute for Innovation, Capacity Building and Sustainability of Agri-Food Production, 5000-801 Vila Real, Portugal; 5 Faculty of Pharmacy, University of Coimbra, 3000-548 Coimbra, Portugal

**Keywords:** zoonosis, *C. burnetii*, prevalence, outbreaks, genotype

## Abstract

**Simple Summary:**

This review article aims to compile the information published in the scientific literature regarding *Coxiella burnetii* infection in goats and their role in the epidemiology of infection, namely their association with the occurrence of Q fever in humans. Q fever presents a worldwide occurrence and the risk that it represents to humans has been recognized since its first description. The characteristics of *C. burnetii* justify its classification as a category B biological threat agent. International public health authorities strongly recommend global monitoring of *C. burnetii*, especially after large-scale Q fever epidemics occurred in The Netherlands, which originated from goat infection. An approach with the characterization of the bacterium, its strategies of infection, and clinical patterns in goats will help to understand the dynamics of infection in an epidemiological analysis and to analyze the role of goats in Q fever.

**Abstract:**

Since its first description in the late 1930s, Q fever has raised many questions. *Coxiella burnetii*, the causative agent, is a zoonotic pathogen affecting a wide range of hosts. This airborne organism leads to an obligate, intracellular lifecycle, during which it multiplies in the mononuclear cells of the immune system and in the trophoblasts of the placenta in pregnant females. Although some issues about *C. burnetii* and its pathogenesis in animals remain unclear, over the years, some experimental studies on Q fever have been conducted in goats given their excretion pattern. Goats play an important role in the epidemiology and economics of *C. burnetii* infections, also being the focus of several epidemiological studies. Additionally, variants of the agent implicated in human long-term disease have been found circulating in goats. The purpose of this review is to summarize the latest research on *C. burnetii* infection and the role played by goats in the transmission of the infection to humans.

## 1. Introduction

The history of Q fever, the disease caused by *Coxiella burnetii*, can be traced back to 1937, when it was described by Edward Holbrooke Derrick in Australia [1]. Almost simultaneously, in the United States, an unknown agent isolated from ticks recovered from Nine Mile Creak region, Montana, was described [2]. Australian and American teams shared their findings and concluded that they were studying the same agent and the same disease [3]. The potential risk of Q fever to public health and the large gaps in the knowledge of this disease were recognized early, namely by the World Health Organization (WHO) that, in 1950, encouraged the epidemiological research. Consequently, Q fever was reported in 51 countries from the five continents [4]. In Europe, Q fever was first reported in Greece, during the Second World War, in German soldiers who had febrile illness, the so-called “Balkan flu” [5].

Nowadays, except in New Zealand, *C. burnetii* is found worldwide, infecting a wide range of domestic and wildlife animals [6,7]. Q fever is listed in the Terrestrial Animal Health Code of the World Organization for Animal Health (WOAH) and all Member Countries are required to report the occurrence of the disease [8].

Since its first report, human Q fever outbreaks have been regularly reported throughout the world [9]. From 2007 until 2010, The Netherlands faced the largest Q fever outbreak ever recorded, resulting in over 4000 reported and 40,000 estimated infected people [10]. This occurrence alerted public health authorities regarding *C. burnetii* and the need for a harmonized monitoring of infection was highlighted [11,12,13,14]. In fact, during the last decade, the number of relevant publications on this subject increased significantly [15].

Despite the wide host range of *C. burnetii*, the infection is mostly recognized in domestic ruminants [7,16,17,18,19]. However, over time, human Q fever outbreaks have often been related to spill-over infection from goats to humans, as shown in Table 1. 

Recently, it was observed that goats played a major role in infecting humans compared with sheep in The Netherlands Q fever outbreak, which was previously associated with small ruminants [18]. Thus, the infection patterns in goats (e.g., dynamics of infection, clinical outcomes, and shedding patterns) need clarification, and experimental studies using goats have improved our scientific knowledge on this topic. Furthermore, the effective strategy of prevention and control of Q fever, in both humans and animals, requires a One Health perspective [28,30]. This review is not to intend to give a global geographic epidemiological view of Q fever, but rather focus on the role of goats in the epidemiology of this zoonotic pathogen. First, the characteristics of the agent and its pathogenesis will be briefly described, considering its significance in understanding the clinical and excretion patterns and followed by the specific infection in goats. Then, the focus will be in the epidemiological role of goats in Q fever dissemination, finally referring to the importance of molecular tools to unravel the complex epidemiology of *C. burnetii*.

This review will focus on *C. burnetii* infection, highlighting the role of goats in the epidemiology of this zoonotic pathogen. An overview of the pathogenesis, as well as of the epidemiological characteristics influencing the dissemination of the infection, will be described referring to the importance of molecular tools to unravel the complex epidemiology of *C. burnetii*.

## 2. *Coxiella burnetii*: The Microorganism and Its Pathogenesis

When Q fever was first described, its causative agent was unknown. In 1948, the genus *Coxiella* was created and *Coxiella burnetii* (Philip, 1948) was listed in the 6th edition of Bergey’s Manual of Determinative Bacteriology [3,33] as the aetiological agent of Q fever.

Phylogenetic investigations based on 16S rRNA sequence analysis placed *C. burnetii* in the gamma group of proteobacteria, belonging to the order Legionellales, family Coxiellaceae, and genus *Coxiella* [34]. The first complete genome sequence of *C. burnetii* was published in 2003. It corresponded to the original strain (RSA 493 strain) firstly isolated from ticks in the United States, also known as the Nine Mile strain. This event led to significant advances in the knowledge of *C. burnetii* [35]. The genome of *C. burnetii* contains conserved genomic regions as well as polymorphic regions [36]. Furthermore, the insertion sequence IS1111 plays an important role in the genomic plasticity of *C. burnetii*. The number of IS1111 elements is highly variable between strains; many different genetic locations are described, showing a direct impact on *C. burnetii* genotypes [37].

*C. burnetii* is a small pleomorphic Gram-negative rod, presenting 0.2–0.4 μm wide and 0.4–1.0 μm long [38]. All the lipopolysaccharides (LPSs) encoding genes are in a 38 Kb region in the *C. burnetii* genome, and it has been observed that mutational variations in this region result in antigenic and virulence shift, termed “phase variation”. Antigenic variation results from an irreversible modification from smooth-type (phase I) to rough-type (phase II) LPS causing a dramatic reduction in virulence [39]. Thus, the avirulent rough LPS (phase II) results from a point/frameshift mutation, small deletion, or transposon insertion in a gene in the LPS biosynthetic pathway [40,41]. Therefore, the sugar composition of phase II LPS is quite different because sugars such as L-virenose dihydrohydroxystreptose and galactosamine uronyl-(1,6) glucosamine are lacking [39,42]. So, the lack of virulence is associated with a shorter LPS and not with a defect in the synthesis of other virulence factors. However, it is interesting to note that avirulent forms of other strains besides Nine Mile show different patterns of deletions/mutations, suggesting that the biosynthesis of LPS in *C. burnetii* is not yet completely understood [40]. The shift from virulent phase I to avirulent phase II is likely due to repeated passages of the strains in cell cultures or embryonated eggs [43].

Phase I *C. burnetii* can be recovered from infected hosts and the smooth-type LPS of phase I disturbs an effective immune response, giving the phase I bacterium the opportunity to survive and multiply in the host cells. Therefore, phase I *C. burnetii* is highly infectious [39]. 

*C. burnetii* exhibits a biphasic developmental cycle in which two main morphological forms are identified: large cell variant (LCV) and small cell variant (SCV) [44]. LCVs have a larger size (>0.5 μm), they are metabolically active, and have less electron dense forms. They have dispersed and filamentous chromatin and possess clearly distinguishable outer and cytoplasmic membranes with exposed LPS on the surface, sharing features with Gram-negative bacteria. These LCVs are sensitive to the decrease in osmotic pressure [45,46,47]. SCVs are small rod-shaped forms ranging typically from 0.2 and 0.5 μm, being filterable through 0.22 μm filters. They are very compact and present low metabolic activity [44,46]. Some structural characteristics of SCVs are the electron-dense and condensed chromatin and the unusual cell envelope characterized by a high number of cross-links in peptidoglycans, which seems to enhance environmental stability [45,48]. Thus, they are very stable in the environment, showing a high resistance to osmotic, mechanical, chemical, heat, and desiccation stresses [44,48].

The primary target cells of *C. burnetii* are blood-circulating monocytes, macrophages (e.g., lymph nodes, spleen, liver, and lungs) [49], and trophoblasts in pregnant females [50].

**Figure 1:**
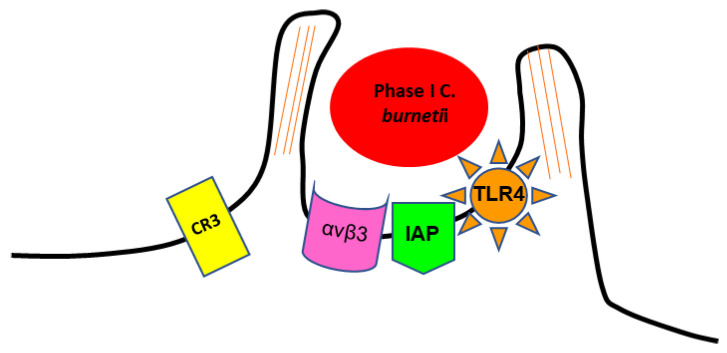
Scheme representing the internalization of phase I SCV of *C. burnetii* by monocyte-like cells.

The internalisation of phase I SCV of *C. burnetii* in target cells involves the recognition of several receptors [51]. It is mediated by the leukocyte response integrin (LRI) (αvβ3) and an integrin-associated protein (IAP) [39,52]. The entry occurs through a microfilament-dependent endocytosis [44,51]. Phase I LPS induces a rearrangement of F-actin cytoskeleton, leading to pronounced membrane protrusions at the site of bacterial adherence. This phenomenon, called membrane ruffling, requires contact between *C. burnetii* and host cells, and depends on the expression of toll-like receptor type 4 (TLR4) on the host cell surface (Figure 1) [53,54,55]. The ability to use αvβ3 integrin for invasion might be exploited by *C. burnetii* as a mechanism to avoid the induction of an inflammatory response, as αvβ3 integrin is typically involved in the removal of apoptotic cells via phagocytosis, being generally associated with an inhibition of inflammation [52]. Thus, *C. burnetii* enters the cells without alerting the immune system [56].

After internalization, bacteria localize within the nascent *Coxiella*-containing vacuole (CCV), which traffics through the endocytic cascade. It develops into an early phagosome acquiring the small GTPase RAB5. This GTPase stimulates the fusion with early endosomes, resulting in acidification of the lumen to approximately pH 5.4 and acquisition of the early-endosomal marker protein 1 (EEA1) [57,58]. Early phagosome is converted into late phagosome acquiring acid hydrolases, which are involved in pronounced degradative activity, which *C. burnetii* can resist [59]. This late phagosome lacks RAB5 and EEA1 but acquires lysosome-associated membrane protein 1, 2, and 3 (LAMP1, LAMP2, and LAMP3) and vacuolar ATPase, which pumps protons into the maturing phagosome to further decrease the luminal pH to about 5.0 [58,60,61]. *C. burnetii* persists and replicates, at a slow rate, within the large CCV with an acidic environment [39,62,63]. The process of phagosome maturation continues with its fusion with lysosomal compartments to acquire cathepsins and hydrolases. The vacuolar ATPase further reduces the pH to around 4.5 [58,64]. Phagosome maturation depends on the balance between pro-inflammatory (IFN-γ, IL-12, and IL-6) and anti-inflammatory (IL-10) cytokines [65]. *C. burnetii* modulates the genesis of CCV and has several strategies for adaptation to the stressful environment. It encodes a significant number of basic proteins that are probably involved in buffering the acidic environment of the CCV. Moreover, four sodium–proton exchangers and transporters for osmoprotectants are codified in its genome, allowing this bacterium to confront osmotic and oxidative stresses [35]. 

During its biogenesis process, CCV becomes large and contains a large number of bacteria [62]. *C. burnetii* does not synthesize its own CCV membrane. Multiple fusion events with autophagosomes along with endolysosomal vacuoles are essential to provide sufficient membrane to enlarge the CCV [66,67]. *C. burnetii* continuously directs fusion with other host cell compartments and inhibits apoptotic cell death, allowing a prolonged infectious cycle [63,68,69,70,71]. 

**Figure 2:**
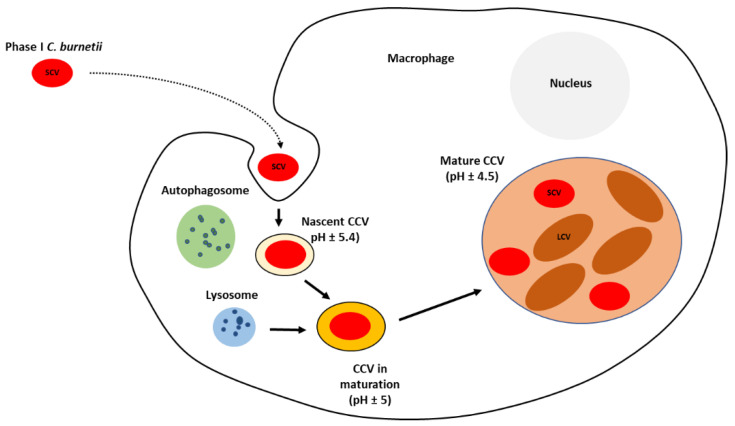
Diagram of the intracellular lifecycle of *C. burnetii*. CCV—*Coxiella*-containing vacuole; SCV—small cell variant; LCV—large cell variant.

The internalised SCV, within the CCV, suffers a differentiation into replicative and metabolically active LCV (Figure 2). The low intra-phagosomal pH and perhaps enzyme system and/or nutrient sources present in the vacuole seem to trigger this differentiation. Lag phase extends to approximately two days post-infection and is composed primarily of SCV to LCV morphogenesis. The exponential phase occurs over the next four days with CCV harbouring replicating LCV almost exclusively. The LCV multiplies and persists within an expanding CCV that contains lysosomal elements, including an acid pH (5.0) and degradative proteases [44,46,59,72]. 

A dramatic expansion of the CCV occurs concomitantly with the appearance of replicating LCV, occupying nearly the entire cytoplasm [44,66]. These metabolically active LCVs also play an important role in cell-to-cell spread during acute infection. This process is facilitated by the display of unique LCV antigens such as a porin protein termed P1. The stationary phase begins six days post-infection, concomitantly with the re-appearance of SCV. Following the accumulation of large numbers of LCVs, *C. burnetii* converts back into SCVs, which are released from heavily infected cells by an undefined mechanism [44].

The resistance properties of these SCVs strongly implicate this form as responsible for long-term extracellular survival and aerosol transmission of *C. burnetii* [44,45].

## 3. Infection and Clinical Outcomes in Goats

It is globally recognized that *C. burnetii* infection occurs mainly by inhalation of contaminated aerosols and, because *C. burnetii* is a highly infective pathogen, low doses cause a high risk of illness [73,74]. So far, experimental studies on goats were not focused on estimating the infectious dose. However, in humans, it was estimated that the 50% infectious dose was around one bacterium [75].

Alveolar macrophages are the first-line defence that confronts *C. burnetii* [49,76]. The ability of these cells to rapidly respond recruiting additional immune cells is central for an effective antibacterial response in early stages of infection [65,77]. In primary infections, after entry into the organism, a bacteraemia occurs, leading to a systemic infection with the involvement of organs such as liver, spleen, lungs, and bone marrow [38]. The organism can subsequently disseminate to colonize and replicate in resident macrophages of different tissues and organs [78]. In pregnant goats, the main target cells are the trophoblasts in the allanthocorion, causing a placentitis and necrosis of placental tissues [79,80]. The amount of *C. burnetii* DNA detected increases until parturition and decreases drastically after parturition, probably by the disappearing of trophoblasts, the replication niche of *C. burnetii* during pregnancy [79,81]. This strong tropism of *C. burnetii* towards placenta does not seem to occur for other tissues of nonpregnant goats and kids, suggesting that pregnant females are more susceptible to *C. burnetii* infection [79,82].

Cell-mediated immunity probably plays a critical role in controlling *C. burnetii* infection [49,55]. Cells belonging to monocyte-macrophage lineage express polarized functional properties. This polarization seems to be closely related to the ability to control *C. burnetii* infection, explaining the bacterial persistence in chronic infections [83]. Classically, M1 polarized macrophages are induced by LPS, IFN-γ, and TNF-α, and participate in the resistance against intracellular pathogens involved in Th1 responses. In contrast, M2-polarized macrophages are induced by IL-4, IL-13, or IL-10 and promote Th2 responses. So, it is thought that the course of infection differs according to the macrophage polarization in response to *C. burnetii* infection [83]. If M1-associated molecules are expressed by macrophages, the bacterial replication will be controlled [62,83], while the stimulation of an M2 response will account for the persistence of *C. burnetii* in macrophages, which become highly permissive to *C. burnetii* replication [83,84,85].

Beyond cell-mediated response, an antibody-mediated immunity also seems to be important in *C. burnetii* infection [49]. Treatment of *C. burnetii* infection with immune sera makes the bacterium more susceptible to phagocytosis and destruction by macrophages [86]. Specific immunoglobulins are secreted following infection [38] and the infection of dendritic cells with antibody-opsonized bacteria results in increased expression of maturation markers and inflammatory cytokines in mice [49]. It can be concluded from field studies that *C. burnetii* antibodies are highly persistent, lasting for several months up to years [87,88]. Thus, both humoral and cellular immunity play a role in *C. burnetii* infection. 

However, the immune control of *C. burnetii* might not lead to its eradication from the infected host [55]. It is also hypothesized that the uterus could be a site of latent infection, hence reactivation during pregnancy can occur [89,90].

In goats, as well as in other domestic ruminants, *C. burnetii* infection often goes unnoticed owing to the absence of symptoms, and the term Coxiellosis is usually used to refer this condition [8]. In the early stages after infection, *C. burnetii* can be detected in the blood, lungs, spleen, and liver. However, it is not clear if its presence in organs other than placenta affects the functions of these organs, as only mild lesions have been described [79,81,91,92]. Experimental infection of non-pregnant goats showed that, at late stages of infection, *C. burnetii* was present in mammary glands, emphasizing the milk as an important shedding route [82]. Infection of pregnant goats may cause a wide range of conditions including abortion, delivery of premature offspring, stillbirth, and weak offspring. Of these, one of the most important outcomes of the *C. burnetii* infection is the abortion, which occur at the end of pregnancy without premonitory signs. In dairy goat herds that experience abortions caused by *C. burnetii*, an increased incidence of metritis can be noticed. Notwithstanding, a clinically normal progeny, which may or may not be congenitally infected, may occur, as described in infection of non-pregnant goats [7,79,82]. However, it seems that apparently healthy kids born from infected mothers may develop respiratory and digestive tract disorders [7]. 

In the season that follows an abortion storm, the multiplication of the organism may be reactivated during pregnancy, leading to reproductive failures [93,94,95]. Even in asymptomatic infections, a latent infection may develop and a reactivation late in pregnancy can occur several days before parturition. Generally, when late-term abortions, stillbirths, or birth of stunted animals are observed in goat flocks, Q fever should be suspected. Usually, up to 90% of the reproductive females within the flock are infected. This is why it is mentioned that *C. burnetii* may cause epidemic herd outbreaks with significant animal losses owing to abortion waves and weak offspring during the parturition period [96,97]. 

## 4. Epidemiological Highlights

*C. burnetii* is a category B biological threat agent because of its impressive stability and resistance, its ability to aerosolize, and its virulence [43,44,45,98]. These characteristics allow the survival of this pathogen in the environment for long periods while keeping its infectivity [45,53]. In fact, viable microorganisms can be recovered after several years in dust, two years at –20 °C, seven to ten months on wool at environment temperature, 150 days in soil, for more than one month on fresh meat, and seven days in water or in milk at room temperature [17,38]. 

The transmission of *C. burnetii* may occur by direct, indirect, or vectorial transmission. The majority of natural *C. burnetii* infections occur by airborne transmission, resulting from the inhalation of aerosolized bacteria [79,93,96]. The resistance of *C. burnetii* allows it to be dispersed by wind far away from its original source. This may cause a long-distance transmission of infection leading to inter-herd transmission of *C. burnetii* or even to dispersion of bacteria to residential locations, causing human outbreaks [99,100,101]. Several studies on human outbreaks report this mid- to long-distance transmission [99,102,103]. Moreover, areas with high wind speed, open landscape, and high temperature increase the risk of infection [104].

Milk is considered a relevant route of bacterial shedding in the infected goats [105] and several studies evidence the presence of *C. burnetii* in goat milk (Table 2). 

Despite the knowledge that *C. burnetii* remains viable in unpasteurized milk, the assumption of the risk of infection by ingestion of *C. burnetii* milk is controversial [108,116,117]. In France, *C. burnetii* was detected in commercially available milk products, but, because its viability was not confirmed, the transmission by consumption of these products was not considered important [118]. Moreover, in a report concerning the public health risks related to raw drinking milk, *C. burnetii* is not mentioned as a biohazard to be transmitted via milk [119]. Thus, for risk assessment, it is assumed that multiplication of the pathogen in milk and milk products does not occur. Furthermore, there are insufficient data for a dose–response model for the oral route in humans [117]. It is known that the pasteurization procedure by ultra-high temperature treatment of milk (72 °C for 15 s) is adequate to eliminate viable *C. burnetii* from whole raw milk [120,121]. Notwithstanding, the risk of infection by consuming unpasteurized milk and raw milk dairy products may not be negligible.

Infected goats also shed high concentrations of *C. burnetii* in placental membranes, birth fluids, and/or abortion products [79,122]. This is an important excretion route in peri-partum period contributing to a high contamination of the environment. Furthermore, *C. burnetii* can also be excreted through faeces and vaginal mucus [79,113]. The shedding is normally very high at the first parturition after the infection, but occasionally, it occurs at subsequent pregnancies accompanied by a considerable number of bacteria excreted through placenta [79,94]. So, normal deliveries in infected females may contribute to the environmental contamination and should, therefore, be considered as a major zoonotic risk [79]. In the environment, bacteria can be easily aerosolized from desiccation of infected placenta and body fluids or from contaminated manure, reaching new hosts [96,101,103].

The risk of infection has been studied at the herd level and at the individual level in prevalence studies by detection of antibodies (Table 3 and Table 4, respectively).

At the herd level, the risk factors for *C. burnetii* infection are as follows: the proximity of an infected farm, the high animal density in a municipality, the high wind speed, an open landscape, the high temperature [123,134,135], the increased size of the herd [123,129,134], the poor hygiene and bio-security measures in the farm, the presence of ticks, the presence of dogs and cats in the farm [134,135], and the presence of swine on farms [123]. At an individual level, it was shown that the risk of positivity increases with age [129].

**Table 4:** Individual seroprevalence of *C. burnetii* in goats.

Country (Area)	Study Period	Type of Sample	Sampling Method	Number of Samples	Test	Cut-Off Value	Prevalence (%)	Reference
Albania	1995–1997	Serum	-	443	ELISA	0.4	8.8	[136]
Bangladesh	2009–2010	Serum	Convenience	529	ELISA	0.4	0.8	[137]
Brazil	2014–2015	Serum	Convenience	312	ELISA	0.4	55.1	[138]
Canada	2010–2012	Serum	Multi-stage random	2195	ELISA	0.4	32.5	[123]
Ethiopia	-	Serum	Multi-stage random	293	ELISA	0.4	35.5	[139]
Great Britain	2008	Serum	Random stratified	522	ELISA	0.4	0.8	[124]
Greece	2014–2015	Serum	Convenience	800	ELISA	0.4	14.4	[140]
India	-	Serum	Convenience	53	ELISA	0.4	5.7	[141]
Iran	-	Serum	Multi-stage random	241	ELISA	0.4	22.4	[142]
Ireland (Republic of)	2005–2007	Serum	Random	590	ELISA	0.4	0.3	[125]
Italy	2012	Serum	Multi-stage random	3185	ELISA	0.4	25.7	[126]
Ivory Coast	2012–2014	Serum	Cluster	622	ELISA	0.4	12.4	[143]
Kenya	2013	Serum	Random	280	ELISA	0.4	18.2	[144]
Lebanon	2014	Serum	Random	384	ELISA	0.4	17.2	[127]
Reunion Island	2011–2012	Serum	Random	134	ELISA	0.4	13.4	[135]
Portugal	2011	Serum	Random	-	ELISA	0.4	10.4	[129]
Spain	2007–2008	Serum	Random	115	ELISA	0.40	8.7	[130]
Spain	2015–2018	Serum	Random	135	ELISA	0.4	24.4	[145]
Switzerland	2011	Serum	Random stratified	321	ELISA	0.4	3.4	[132]
The Gambia	2012	Serum	Multi-stage random	484	ELISA	0.4	24.2	[112]
The Netherlands	2008	Serum	Random	3134	ELISA	0.4	7.8	[133]
USA	2012–2014	Serum	Random	608	ELISA	0.4	3.8	[115]
Vietnam	2016–2017	Serum	Random	1458	ELISA	0.4	4.1	[146]

The number of Q fever cases varies geographically, and a seasonal variation is also described [100]. In the Northern Hemisphere, acute Q fever cases are more often reported in spring and early summer, showing a slow rise in reported cases in March and April, probably associated with the start of lambing/kidding, and the main peak occurs between May and July [27,147,148]. This occurs probably because of the “outside” lambing/kidding during spring associated with heavy environmental contamination with *C. burnetii*. It is known that the lambing season in October is not related to a higher incidence in humans, which might be owing to “indoor” lambing [96], which is also consistent with the study conducted in the South of France, showing that autumn is not a very windy season, which might explain the lower incidence of human Q fever at this time of the year [99]. 

**Figure 3:**
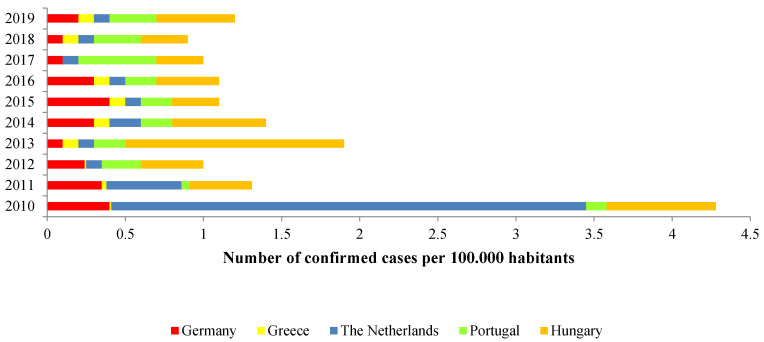
Rates of confirmed human cases of Q fever in four European Countries from 2010 to 2019 [148,149,150].

In most European countries, Q fever cases in humans and animals are reported regularly [8,148]. In humans, after the largest ever recorded outbreak in The Netherlands, the number of notified cases has suffered in general a sustained decrease in Europe and, nowadays, small outbreaks still occur, as shown on Figure 3, mainly in areas with infected livestock herds [148,149,150].

**Figure 4:**
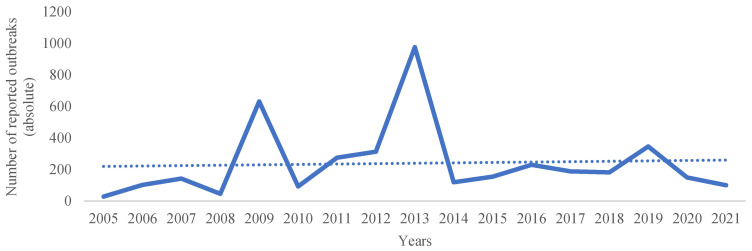
Number of Q fever outbreaks in goats reported in European countries from 2005 to 2021 and linear trend line [8].

Regarding the report of infection in goats, the WAHIS Interface from the World Organization for Animal Health (WOAH) indicates the number of outbreaks reported in each country per year, which is systematized for European countries in the Figure 4. However, the analysis must be performed carefully because it is based on a notification procedure, thus it may not indicate the true prevalence as differences in the procedure of notification may differ between countries. Thus, data from epidemiological studies (Table 3 and Table 4) can be more reliable within the reality in a region/country.

*C. burnetii* infection in humans is usually considered an occupational threat. Some professional groups are more prone to exposure with *C. burnetii*. For instance, the farming workforce constitutes a relevant occupational risk group because of their contact with infected livestock, namely during breeding practices [151,152]. Moreover, veterinarians, laboratory workers, and abattoir workers are also at risk of being infected [8,153,154,155,156], as well as workers in the wool, tanneries, fur, meat, leather, and timber industries [152].

However, community outbreaks have been described very often. Factors as living on rural or sub urban areas [133] and in the proximity of positive farms [157] substantially increase the risk of Q fever.

The danger of *C. burnetii* being used as a biological weapon is a global concern. The low infectious dose for humans and its ability to spread over large distances on the wind would cause an enormous impact on human health. Additionally, wide-scale consequences would occur because of animal infection (i.e., domesticated, and wild animals) that could represent secondary sources of infection for humans [98] and even compromise agriculture and food production. 

## 5. Molecular Epidemiology: An Added Value

Nowadays, molecular epidemiology is crucial in monitoring programs of *C. burnetii* and in investigation of Q fever outbreaks. The genetic heterogeneity of *C. burnetii* can be assessed by several molecular techniques. Today, the most adopted methods to define phylogeny are the multi loci variable-number tandem repeat analysis (MLVA) and the multispacer sequence typing (MST) [96,158,159]. A large number of MLVA data exist for European countries, even if the lack of consensus between scientists hampers comparison. Overall, a common pool of MLVA genotypes is present in Europe, together with novel genotypes sporadically found in specific countries [160,161,162,163]. Different MLVA genotypes could correspond to an identical MST type, indicating the MLVA method as more discriminatory than MST. Furthermore, the availability of a free-access database on the internet increased the interest in these methods to characterize *C. burnetii* strains circulating in a region in a normal context or in the case of outbreak [106,164,165]. 

A systematic genotyping provides a descriptive database, enabling to monitor the temporal and geographical evolution of strains, thus helping to trace the origins of the outbreaks and to identify interspecies transmission. These data can help to explain different scenarios of dissemination and contribute to finding efficient control measures [37,159,165,166]. For instance, in Portugal, the involvement of different genotypes in acute and in long-term infections of Q fever was found, but only one genotype found in long-term Q fever was linked to the one identified in goats from the same region [161]. Moreover, in Belgium, an emerging CbNL01-like genotype was identified in goats and this strain was isolated from half of the field samples isolated from the Dutch outbreak. However, no impact on the number of human cases was observed [106]. In Poland, examination of nine *C. burnetii* samples from goats revealed the presence of three MLVA genotypes (I, J, and PL1) and one sequence type (ST61). These MLVA and MST profiles were different to the strains’ profiles involved in the Q fever outbreak in the Netherlands [109]. Table 5 shows the *C. burnettii* genotypes identified by MST in goats and in other hosts (human, ruminants, and vectors), showing the wide transmission of specific genotypes.

An analysis of MLVA and MST genotypes published in international databases showed that human isolates of *C. burnetii* frequently belong to the same genomic group of caprine isolates [168]. Furthermore, a geographical niche for *C. burnetii* genotypes was demonstrated. Although some isolates present a worldwide distribution, others show a geographic localization. Some genomic groups occur predominantly in Northern and Central Asia, Eastern and Central Europe, and Africa, while others are found mainly in central Europe or even in other areas [168]. For instance, MST8 reported in goats from Spain (Table 5) was the most found genotype in goat milk in the United States [169].

Thus, genotyping demonstrates that isolates in human Q fever are frequently genetically related to isolates circulating in goat populations, which reinforces the role of goats as important sources of infection for human populations.

## 6. Conclusions

In conclusion, *C. burnetii* presents a wide host range; however, it is mostly recognized in domestic ruminants. Q fever is not very commonly diagnosed in humans, in part because primary infection is frequently asymptomatic. The development of molecular tools has allowed to unravel the dynamic of genotypes’ circulation. Genotyping of *C. burnetii* indicates that, often, infected goats are the source of infection in human Q fever outbreaks. The pathogenesis is complex and not entirely understood at the host level, which highlights the requirement of future research to grasp the dynamics of the infections in specific hosts, as well as to unravel strain characteristics that may determine its virulence, affect the course of the disease and the clinical outcome, or influence the affinity to specific hosts. Yet, the association of genotypes with high virulence or host specificity remains to be demonstrated. An international genotype database facilitates the identification of emerging genotypes and their epidemiological features, also relying on a standard molecular method, which allows interlaboratory comparison. Thus, a worldwide surveillance in goats based on molecular epidemiology will be an important strategy to effective control of this zoonotic and highly resistant pathogen that fits in the One Health approach that considers animals, environment, and humans.

## Figures and Tables

**Table 1:** Human Q fever outbreaks associated with goats.

Country (Area)	Period	Reference
Australia	2012–2014	[20]
Bulgaria	2004	[21]
	2007–2011	[22]
China	2018–2019	[23]
France	-	[24]
	2007	[25]
Newfoundland	1999	[26]
Slovakia	1993	[27]
The Netherlands	2007–2020	[28,29]
United Kingdom	1987	[30]
USA	-	[31]
	2011	[32]

**Table 2:** *C. burnetii* DNA detected in milk samples from goat herds.

Country (Area)	Study Period	Type of Sample	Number of Samples	Test	Prevalence (%)	Reference
Belgium	2009–2013	BTM ^a^	1924	Real-time PCR	12.1	[106]
France	-	BTM ^a^	120	PCR	19.0	[105]
Iran	2008	BTM ^a^	110	Nested PCR	4.5	[107]
Italy	2018–2020	Milk	68	PCR	25.0	[108]
		Cheese	15	PCR	6.7	
Poland	-	BTM ^a^	35	Real-time PCR	54.3	[109]
Portugal	2009–2013	BTM ^a^	12	Real-time PCR	0.0	[110]
Switzerland	2006	Milk	39	Nested PCR	0.0	[111]
The Gambia	2012	Milk	33	PCR	2.94	[112]
The Netherlands	2008	BTM ^a^	292	Real-time PCR	32.9	[113]
Turkey	-	Milk	50	PCR	4.0	[114]
USA	2012	Milk	387	Real-time PCR	2.5	[115]

^a^ BTM—bulk tank milk.

**Table 3:** Prevalence of antibodies anti-*C. burnetii* at goat herd level.

Country (Area)	Study Period	Type of Sample	Sampling Method	Number	Test	Cut-Off Value	Prevalence (%)	Reference
Canada	2010–2012	Serum	Multi-stage random	76	ELISA	0.4	63.2	[123]
Great Britain	2008	Serum	Random stratified	145	ELISA	0.4	3.0	[124]
Ireland (Republic of)	2005–2007	Serum	Random	66	ELISA	0.4	1.5	[125]
Italy	2012	Serum	Multi-stage random	206	ELISA	0.4	19.5	[126]
Lebanon	2014	Serum	Random	128	ELISA	0.4	45.3	[127]
Norway	2009	BTM ^a^	Random	348	ELISA	0.4	0	[128]
Portugal	2011	Serum	Random	52	ELISA	0.30	28.8	[129]
Spain	2007–2008	Serum	Random	11	ELISA	0.40	45.0	[130]
Sweden	2010	BTM ^a^	Random	58	ELISA	0.4	1.7	[131]
Switzerland	2011	Serum	Random stratified	72	ELISA	0.4	11.1	[132]
The Netherlands	2008	Serum	Random	442	ELISA	0.4	17.9	[133]
USA	2012–2014	Serum	Random	89	ELISA	0.4	11.5	[115]

^a^ BTM—bulk tank milk.

**Table 5:** *Coxiella burnetii* genotypes identified in goats and other hosts using multispacer sequence typing (MST).

MST	Species	Country	Reference
8	Goat	Spain	[160,167]
	Sheep	Spain	
	Human	Portugal, France, and USA	
13	Goat	Portugal, Spain	[160,167]
	Sheep	Spain	
	Cattle	Spain	
	Human	Portugal	
	Ticks	France	
18	Goat	Germany, Spain	[160,167]
	Sheep	Germany	
	Cattle	Poland	
	Human	France, Greece, Italy, Poland, Slovakia, and Romania	
30	Goat	Namibia	[167]
32	Goat	Austria	[167]
	Human	France and Germany	
53	Goat	France	[167]
58	Goat	Libano	[167]
61	Goat	Poland	[167]
	Cattle	Iran	[167]
62	Goat	Iran	[167]
	Sheep		[167]
	Cattle		[167]
66 to 70	Goat	Thailand	[167]
74	Goat	Brazil	[167]
	Cattle		[167]

## Data Availability

Publicly available datasets were analyzed in this study. This data can be found here: WAHIS Interface. Available online: https://wahis.woah.org/#/home and Database on Multi Spacers Typing of *Coxiella burnetii*. Available online: https://ifr48.timone.univ-mrs.fr/mst/coxiella_burnetii/.
